# Visualization of Critical Limits and Critical Values Facilitates Interpretation

**DOI:** 10.3390/diagnostics15050604

**Published:** 2025-03-02

**Authors:** Ania Shah, Jenna Dohner, Kaifeng Cheng, Maria Garcia, Gerald J. Kost

**Affiliations:** 1 University of California, Davis, CA 95616, USA; aamshah@ucdavis.edu (A.S.); kfcheng@ucdavis.edu (K.C.); 2 University Honors Program, University of California, Davis, CA 95616, USA; jennadohner@gmail.com (J.D.); mbegarcia@ucdavis.edu (M.G.); 3 Pathology and Laboratory Medicine, School of Medicine, University of California, Davis, CA 95616, USA

**Keywords:** critical limits, critical values, visualization, point-of-care testing

## Abstract

**Background/Objectives:** This study aimed to analyze critical limit and critical value test lists from major US medical centers, identify changes in quantitative critical limit thresholds since 1990, document notification priorities for qualitative and new listings, and visualize information alongside clinical thresholds and pathophysiological trends. **Methods:** A systematic search was conducted, acquiring 50 lists of critical limits and critical values from university hospitals, Level 1 trauma centers, centers of excellence, and high-performing hospitals across the US. Lists were obtained through direct contact or web-accessible postings. Statistical analysis used the Kruskal–Wallis non-parametric test and Student’s *t*-test to determine significant differences between 1990 and 2024 critical limits. **Results:** Statistically significant differences were identified in various clinical tests between 1990 and 2024, comprising glucose, calcium, magnesium, CO_2_ content, blood gas parameters, hematology, and coagulation tests. Ranges for critical limits narrowed for several tests, and new measurands were added. Cardiac biomarkers were infrequently listed. Point-of-care testing (POCT) listings were sparse and showed significant differences from main lab values in the high median critical limit for glucose **Conclusions:** Visualizing this information has potential benefits, including ease of interpretation, which can improve patient care, reduce inconsistencies, and enhance the efficiency and quality of healthcare delivery.

## 1. Introduction

The urgent communication of abnormal test results is a critical aspect of clinical care, ensuring timely and potentially life-saving interventions. This practice, which began decades ago with the use of lists of critical limits and critical values to trigger clinical warnings, has evolved significantly over the years. The first national surveys published in *Journal of the American Medical Association (JAMA)*, *Pediatrics, Archives of Pathology and Laboratory Medicine*, and *Medical Laboratory Observer (MLO)* between 1990 and 1993 codified this important practice in the United States [1,2,3,4]. Recent summaries of critical limits and critical values for adults have been annually compiled in the MLO Clinical Laboratory Reference (CLR) from 1992 through 2025 [5].

Our goals are to report the findings of a comprehensive search for critical limit and critical value test lists used by major US medical centers, identify changes in quantitative critical limit thresholds since 1990, and document notification priorities for qualitative and new listings. Institutions adjust quantitative critical limits and select qualitative critical values to identify extremely abnormal findings that trigger life-saving treatment. Caregivers then use critical notifications to facilitate rapid response and therapeutic decisions at the bedside, in emergency rooms, and when performing POCT. Accreditation agencies, such as the US Joint Commission, require hospitals to create and maintain policies for the urgent notification of critical test results.

By examining the evolution of critical limits and values between 1990 and 2024 and by visualizing critical decision thresholds and professional recommendations, we aim to enhance accessibility and clarity of diagnostic criteria for critical conditions and better illustrate observed changes. Visualization of critical limits may also benefit expeditious decision-making and increasing quality of patient care [6,7,8].

## 2. Materials and Methods

### 2.1. Definitions

A critical limit is defined as a low or high quantitative threshold of a life-threatening diagnostic test result. A critical value is defined as a qualitative result (such as a positive COVID-19 rapid antigen test) warranting urgent notification. Both demand rapid response, and potentially life-saving treatment, isolation of the patient, or other timely medical interventions [9,10].

### 2.2. Acquiring Lists

Lists of critical limits and critical values were acquired from university hospitals, Level 1 trauma centers, centers of excellence, and high-performing hospitals. Centers of excellence have specialized programs within healthcare institutions known for their high levels of expertise and resources, focusing on specific medical areas and providing comprehensive care [11,12]. High performing hospitals were defined as those ranked in the top ten percent nationally in at least three metrics according to the US News & World Report’s Best Hospitals ranking. These rankings are based on metrics including factors such as positive organizational culture, adaptable and attentive senior management, effective performance monitoring, competent workforce, exceptional leadership, expertise-driven practice, and interdisciplinary network [12]. Medical centers were selected across four US Census Bureau regions: Northeast, Midwest, South, and West. These regions were further divided into nine census divisions (New England, Middle Atlantic, East North Central, West North Central, South Atlantic, East South Central, West South Central, Mountain, and Pacific). Notification lists and policies were obtained through direct contact and web-accessible postings [13]. Initial contact with potential hospitals was made via email with the IRB approval number, followed by phone calls using a standardized interview guide.

PubMed articles and internet postings were identified using browser keywords such as “critical limits”, “critical values”, “critical-risk results”, “significant-risk results”, “alert values”, “hypercritical results”, and “panic values”. Multiple search hits zeroed in on the same hospital documents, providing assurance that none were missed. Results from institutions that fell within the inclusion criteria outlined previously were included in the final compilation of critical notifications lists examined.

### 2.3. POCT Specific Methods

We analyzed the *n* = 50 dataset of critical notifications lists and identified the listings specific to POCT. 13 hospitals’ POCT listings were either located in dedicated sections within these lists or embedded throughout. A list serve posted to the Association of Diagnostic and Laboratory Medicine yielded listings from 4 additional hospitals, bringing the total to 17.

### 2.4. Databases

Raw data from the 1990–1993 US National Surveys archived by Knowledge Optimization were used for comparison with our findings. Changes in raw data from 1990 to 1993 were compared with web-listed and directly collected raw data in 2024.

### 2.5. Changes over Time

Changes in quantitative critical limits were determined by comparing the means and medians of quantitative critical limits in 1990–1993 national surveys to the lists found on the internet or shared directly. In addition to clinical chemistry, hematology, and other laboratory disciplines, notifications studied in detail included cardiac biomarkers—for their importance in emergency and crisis care- and highly infectious diseases to update actionable tests used during the pandemic.

### 2.6. Statistical Analysis

The Shapiro–Wilk Test was used to determine the normality of the data, which was confirmed by inspecting histograms. Most quantitative critical limits did not have a normal distribution. Therefore, we primarily used the Kruskal–Wallis non-parametric test to determine if differences over three decades were statistically significant. Differences were considered significant if *p* < 0.05 (denoted by *), or if *p* < 0.01, highly significant 01 (denoted by **). Cell entries in the tables are boldface when significantly different. Please note that the Kruskal–Wallis test does not directly compare medians, but instead the raw distributions of data and therefore can generate a significant *p*-value even if two medians are equal. In the case where both 1990 and 2024 data were normally distributed, significant differences were determined using Student’s *t*-test for means with unequal variances.

The order of the measurands in the tables was determined from frequencies of the 2024 listings. Listing frequencies were determined by dividing by 50 (2024 listings), 92 (1990 adult national survey [1]), or 100 (1993 national survey of ionized calcium critical limits [3]).

### 2.7. Decision Thresholds and Professional Recommendations

Decision thresholds and professional recommendations were identified by reviewing recent guidelines by organizations such as the American Diabetes Association (ADA) and peer-reviewed articles. Recommendations made by government agencies including the Centers for Disease Control and Prevention (CDC), National Institute of Health (NIH), World Health Organization (WHO), and Infectious Disease Society of America (IDSA) for qualitative critical values were also considered.

### 2.8. Ethics

The UC Davis IRB deemed this research exempt (ID 2078118-1). Reporting of notification lists and research results is anonymous.

## 3. Results

The median number of tests on each critical notifications lists was 72. Significant differences (*p* < 0.05) were observed in chemistry (glucose, calcium, magnesium, and CO_2_ content), blood gas (arterial pCO_2_ and arterial pO_2_), hematology and coagulation (platelets, partial thromboplastin time, white blood cell count, and prothrombin time), newborns (glucose, potassium, hematocrit, and arterial pO_2_), and point-of-care (glucose). Chemistry, blood gas, and hematology and coagulation listings were expanded from 1990.

### 3.1. Chemistry Tests

Table 1 summarizes statistically significant differences in chemistry tests between 1990 and 2024. Significant changes were observed in glucose (low median critical limit, *p* < 0.01), calcium (low and high median critical limit, *p* < 0.01), magnesium (high critical limit, *p* < 0.01), and CO_2_ content (high critical limit, *p* < 0.01). Chemistry tests also expanded to include ammonia.

**Figure 1:**
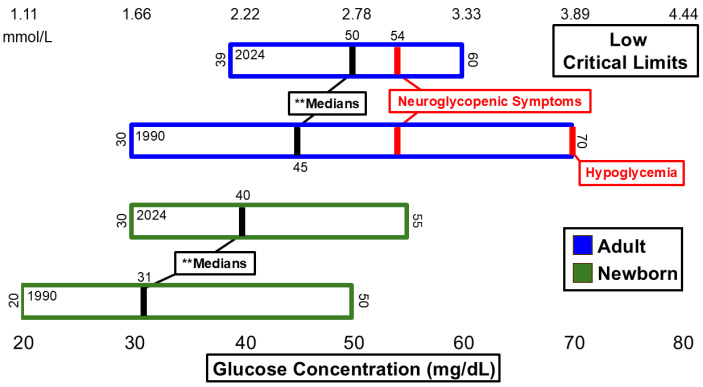
Glucose low critical limits. ** Significant difference in medians at *p* <0.01.

**Figure 2:**
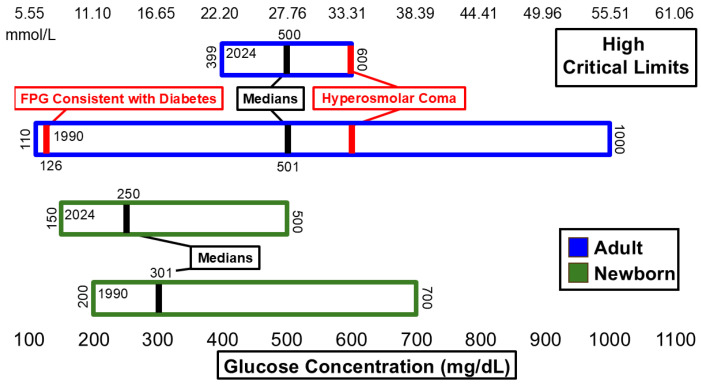
Glucose high critical limits.

Figure 1 and Figure 2 summarize significant changes in glucose. The low median critical limit for adult glucose (50 mg/dL) remains below the 54 mg/dL threshold for neuroglycopenic symptoms as recommended by the American Diabetes Association (ADA) [14] and is still below the threshold for hypoglycemia [14]. A total of 6 hospitals of the *n* = 54 dataset reported low critical limits of 54 mg/dL. Six additional hospitals listed low critical limits of 55 mg/dL or higher. The high critical limit for glucose (500 mg/dL) is above the 110 mg/dL threshold for fasting plasma glucose measurement consistent with diabetes [15] and lower than the 600 mg/dL threshold associated with hyperosmolar coma [16]. The ranges for both low and high critical limits for adults narrowed.

### 3.2. Blood Gas Tests

Statistically significant differences in blood gas tests are summarized in Table 2. Significant differences (*p* < 0.05) observed in blood gas tests included the low median critical limits for arterial pCO_2_ and arterial pO_2_. It should be noted that significant differences were identified in the raw data that caused dissimilar histograms. Compared to 1990’s national surveys, blood gas measurands expanded to include carboxyhemoglobin, methemoglobin, venous pCO_2_, and venous pH.

**Figure 3:**
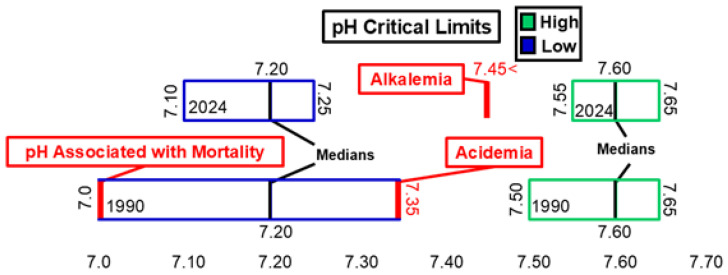
pH low and high critical limits.

**Figure 4:**
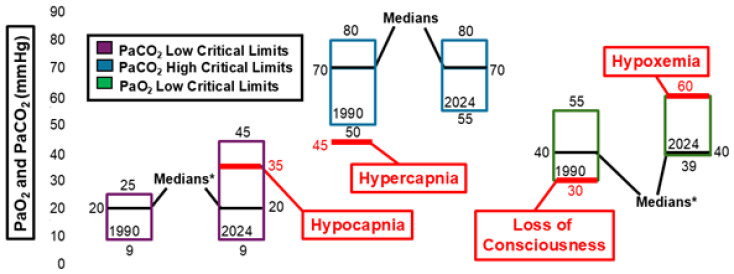
Arterial pCO_2_ (PaCO_2_) and arterial pO_2_ (PaO_2_) critical limits. * Significant difference in medians at *p*< 0.05.

Figure 3 and Figure 4 illustrate changes in arterial pH, arterial pCO_2_, and arterial pO_2_ critical values. Between 1990 and 2024, the ranges for both low and high critical limits for arterial pH narrowed. However, neither the low nor high critical limit for arterial pH showed significant differences during this time frame. The median critical limits for low and high arterial pH remained consistent across 1990 and 2024. The low median critical limit was higher than the 7.0 pH threshold associated with mortality [17] and lower than the 7.35 pH threshold associated with acidemia [18]. The high median critical limit was higher than the 7.45 pH threshold associated with alkalemia [18]. The low and high median critical limits for arterial pCO_2_ and low median critical limit for arterial pO_2_ did not change between 1990 and 2024. However, the distribution of raw data for these critical limits did change significantly within this timeframe. The low critical limit for arterial pCO_2_ (20 mmHg) was lower than the 35mmHg threshold associated with hypocapnia [19]. The high median critical limit for arterial pCO_2_ (70 mmHg) was higher than the 45 mmHg threshold for hypercapnia [20]. The low median critical limit for arterial pO_2_ (40 mmHg) was higher than the 30 mmHg measurement associated with loss of consciousness [20] and lower than the 60 mmHg threshold associated with hypoxemia [20].

### 3.3. Hematology and Coagulation Tests

Table 3 summarizes statistically significant differences identified in hematology and coagulation. Significant changes were seen in the following measurands: platelets (low median critical limit, *p* < 0.01), partial thromboplastin time (PTT) (high median critical limit, *p* < 0.01), white blood cell (WBC) count (low and high median critical limits, *p* < 0.01), and prothrombin time (high mean critical limit, *p* < 0.01). Hematology and coagulation listings also expanded to include absolute neutrophil count (ANC), band count, blast cells, international normalized ratio (INR), and WBC count in cerebrospinal fluid (CSF).

**Figure 5:**
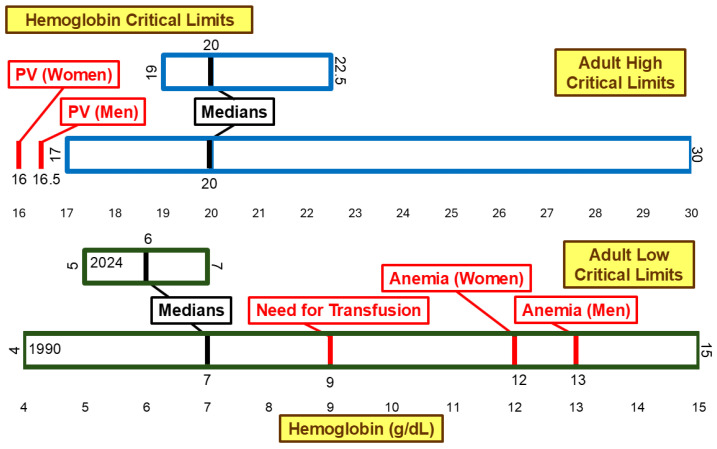
Hemoglobin low and high critical limits. PV, Polycythemia Vera.

Figure 5 illustrates hemoglobin critical limits. Neither the low median critical limit nor the high median critical limit changed significantly between 1990 and 2024. The low median critical limit changed from 7 g/dL to 6 g/dL, and the range narrowed from 4 g/dL–15 g/dL in 1990 to 5 g/dL–7 g/dL in 2024. This is a shift away from the following clinical thresholds: 9 g/dL associated with a need for transfusion [21], 12 g/dL associated with anemia in women [21,22], and 13 g/dL associated with anemia in men [21,22]. The high critical limit range narrowed from 17 g/dL–30 g/dL to 19 g/dL–22.5 g/dL. However, the medians remained 20 g/dL. This is higher than the 16 g/dL and 16.5 g/dL [23,24,25] thresholds associated with the diagnosis of polycythemia vera in women and men, respectively.

**Figure 6:**
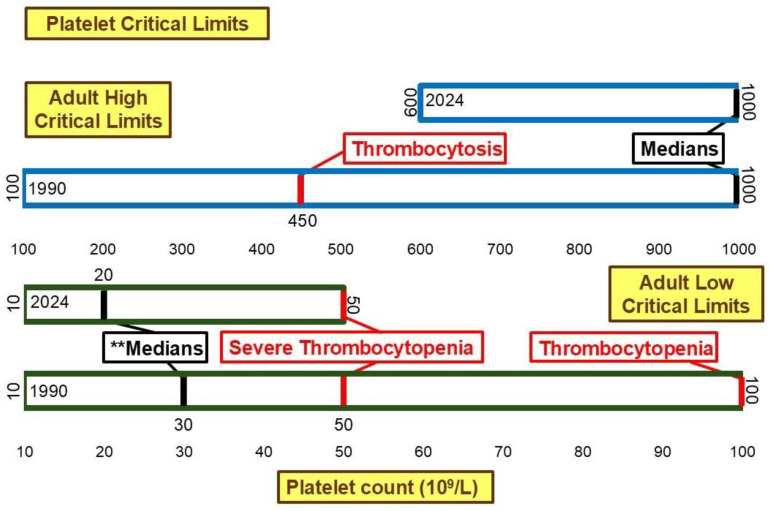
Platelet low and high critical limits. ** Significant difference in medians at *p* <0.01.

Figure 6 illustrates the critical limits for platelet count. The low median critical limit changed significantly (*p* < 0.01) from 30 × 10^9^/L to 20 × 10^9^/L between 1990 and 2024. This shifted the low median critical limit further away from the 50 × 10^9^/L [26] and 100 × 10^9^/L [26] thresholds associated with severe thrombocytopenia and thrombocytopenia, respectively. While the range for high critical limit narrowed from 100 × 10^9^/L–1000 × 10^9^/L to 600 × 10^9^/L–1000 × 10^9^/L, the median high critical limit remained 1000 × 10^9^/L. This is higher than the 450 × 10^9^/L [27] threshold associated with thrombocytosis.

### 3.4. Newborn Critical Limits

Table 4 summarizes statistically significant differences identified in newborn tests including: glucose (low median critical limit, *p* < 0.01), potassium (low and high median critical limits, *p* < 0.01), hematocrit (low median and high mean critical limits, *p* < 0.05), and arterial pO_2_ (low median critical limit, *p* < 0.05).

### 3.5. Cardiac Biomarkers

**Figure 7:**
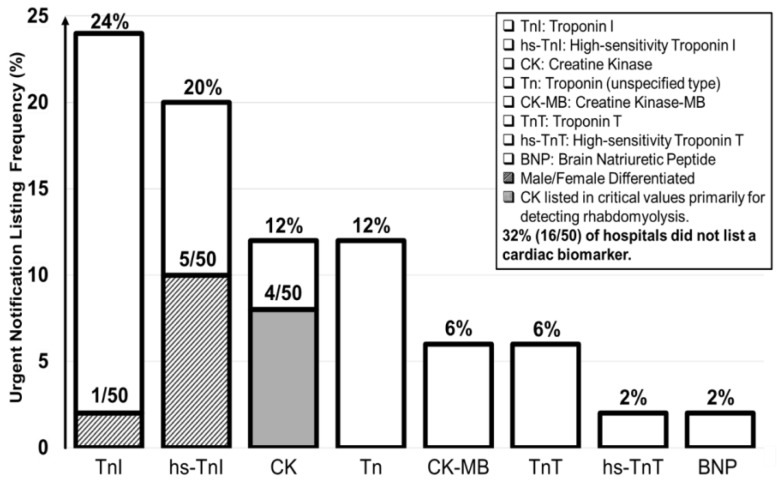
Cardiac biomarker listings.

Table 5 summarizes listings of cardiac biomarkers. 34 hospitals listed cardiac biomarkers: Troponin I (TnI) (12), high-sensitivity Troponin I (hs-TnI) (10), Creatine Kinase (CK) (6), Troponin (6), Creatine Kinase MB (3), Troponin T (3), high-sensitivity Troponin (2), Brain Natriuretic Peptide (1), and high-sensitivity Troponin T (1). Among the cardiac biomarkers, TnI and hs-TnI were listed most frequently, while 16 hospitals listed no cardiac biomarkers at all. CK was listed by six hospitals, with four using it to diagnose rhabdomyolysis. Figure 7 illustrates listings of cardiac biomarkers.

### 3.6. Ranges and Median Numbers of Tests

**Figure 8:**
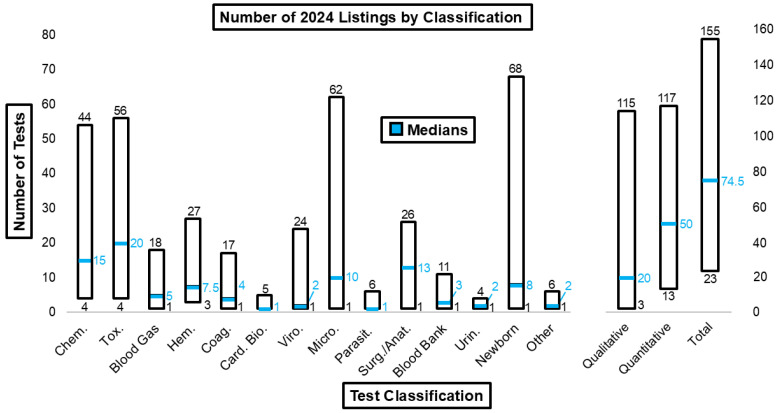
Number of 2024 listings by test classification.

Figure 8 illustrates the ranges and median number of tests for each laboratory discipline. The ranges for listed chemistry, toxicology, microbiology, newborn, qualitative, quantitative, and total tests were especially wide.

### 3.7. Bioterrorism Threats and Pathogens

Table 6 summarizes pathogens that most frequently appear as critical values. The figure also highlights pathogens deemed to warrant urgent notification by the NIH [28], CDC [29], WHO [30], and IDSA [31].

### 3.8. Critical Values

Table 7 summarizes critical values identified in 2024. Additional frequent critical values included: positive culture (82%) or Gram stain (60%) from sterile body fluid, positive Acid-Fast Bacillus (AFB) (68%), and pathogenic fungus in invasive space (44%).

### 3.9. POCT

We acquired POCT listings from 17 institutions. These results are summarized in Table 8. Only the high median critical limit showed a significant difference between POCT and main lab glucose measurements.

## 4. Discussion

Medical centers rely on multiple sources to determine appropriate critical limits and critical values for their institutions. Sources may include national surveys, such as the one published in MLO CLR in 2024 [5] and studies conducted by the institutions themselves regarding the effectiveness of current critical notification thresholds [32] and the potential effect of changing critical limits on call volume and patient outcomes [33].

Only 3 pathogens listed in the *n* = 50 dataset—*Vibrio cholerae,* COVID-19, and the Influenza viruses—were deemed urgent notifications by the CDC, NIH, WHO, and IDSA, but were listed by only 4% of the institutions in our survey. Collaboration between government agencies and medical centers may help identify a more comprehensive and concise list of pathogens that warrant immediate intervention [34]. Establishing standardized lists of pathogens that are universally deemed as warranting urgent notification may help ensure consistency across different institutions, further enhancing standards of patient care [9]. Hospitals could establish standard formats for web-based postings of critical limits and critical values. Web-based postings would allow hospitals to better assess critical limit and critical value notification lists and policies across the country and set the stage for future harmonization [35,36].

Tests displaying significant changes between 1990 and 2024, such as glucose (adults and newborns), total calcium, magnesium, CO_2_ content, platelet count, partial thromboplastin time (PTT), white cell count, prothrombin time (PT), and potassium (newborns), were among the top 15 tests associated with patient death within 24 hours of reporting test results [37].

Although the low median critical limit for blood glucose increased significantly between 1990 and 2024, it remains lower than the ADA-recommended threshold for neuroglycopenic symptoms. This may be because hospitals are basing their critical limits on factors such as laboratory alerts and call volume [38]. This highlights the need for ongoing evaluation and updating of critical notifications lists in healthcare institutions. Adopting standardized approaches for identifying and treating pathophysiological trends can enhance consistency and timeliness, thereby improving patient care [10].

The ranges for arterial pCO_2_ low and high critical limits and arterial pO_2_ low critical limits showed significant changes, although the median critical limits for these measurands did not change. This lack of consensus across institutions indicates uncertainty and may result in patients being treated with different standards of care. This variability is not consistent with the national standard of care [39]. For hospitals whose listed critical limits for these measurands fall below or above the median (for low and high critical limits, respectively), there is an added risk of interventions being enacted too late.

The median low critical limits for hemoglobin and platelet count have shifted away from thresholds associated with blood transfusion needs and severe thrombocytopenia in both men and women. Some institutions may set lower critical limits to reduce unnecessary transfusions and their associated risks [40,41,42]. However, shifting critical limits away from severe clinical thresholds, while decreasing risk for some patients, may delay detection of life-threatening conditions and the delivery of life-saving care for others [43]. Institutions could adopt less conservative critical limits, further from the clinical thresholds such as those identified in Figure 5 and Figure 6, by implementing specific policies that outline testing and critical values for transfusion need indicators prior to major events, such as surgery [21].

Troponin I and High-sensitivity Troponin I are crucial for the timely diagnosis of acute myocardial infarctions (AMIs) and other cardiac conditions due to their high specificity for cardiac muscle damage [43]. The omission of cardiac biomarkers from critical notifications lists entirely can lead to delays in diagnosing and treating cardiac emergencies [44]. Institutions without cardiac biomarkers listed should consider adding hs-TnI to their critical notifications lists due to its sensitivity and cost-effectiveness [45].

While six hospitals listed CK as a cardiac biomarker, four hospitals used it specifically to diagnose rhabdomyolysis. CK is less sensitive than other listed tests, such as troponin, in detecting cardiac muscle damage [46]. Reserving CK as a tool for monitoring muscle damage, such as in cases of rhabdomyolysis, is recommended due to its sensitivity and specificity in indicating severe muscle damage [47,48].

The variability in the number of listed tests across different categories, including chemistry, toxicology, microbiology, newborn screening, and both qualitative and quantitative tests, is notably wide. This wide range can lead to inconsistencies in patient care and inefficiencies in treatment delivery [49]. Collaboration between medical centers aimed at reaching a consensus on standardized lists of tests could significantly enhance efficiency, cost-effectiveness, and the overall quality of patient care [9].

COVID-19 is listed as a critical value by only two hospitals. This is a significant omission, as urgent notification of this pandemic threat can enhance awareness, accelerate infection control, and mitigate contagion [50,51]. Institutions should monitor new notifications, such as positive COVID-19 test results, to speed up infection control in the event of future pandemic threats.

Analysis of our dataset found that, with only six exceptions, urgent notification lists specific to POCT were not present. This finding is noteworthy and contrary to the global expansion of POCT for COVID-19 detection following the pandemic [52]. The absence of dedicated POCT notifications lists may increase liability due to delays in therapeutic care after detection of critical results at the bedside [53]. Furthermore, there is a risk that actionable bedside critical results may not be consistently communicated to the appropriate healthcare professionals who can assess and treat patients effectively [51]. Delineating POCT into concise, separate lists could mitigate these issues and contribute to cost-effectiveness by streamlining the testing and notification process [54]. Clear documentation of policy surrounding collecting and reporting POC values will ensure patient safety and the institution’s compliance with accreditation standards [55].

The use of visual logistics in representing significant changes in qualitative tests will help contextualize critical limits against professional recommendations [8], allowing identification of discrepancies in what national organizations recommend. Visual logistics can also substantially increase the accessibility and clarity of this information and serve as an educational tool for health personnel [56]. The implementation of visual logistics may lead to faster communication and thereby improve patient care by allowing healthcare providers to quickly identify and respond to critical changes [57].

There are limitations to our study. The sample of seventeen institutions with POCT listings represented all that were accessible online, with the addition of comprehensive efforts to acquire additional lists. Furthermore, our results include tests such as INR and Troponin, that were not present in the 1990 datasets, as indicated in Table 3, Table 4 and Table 5 and Figure 7. Several new qualitative listings and infectious threats were found. However, we did not contact hospital staff to determine why they were listed.

## 5. Conclusions

These findings underscore the importance of regularly updating and standardizing critical limits and critical values lists to align with decision thresholds. The visualization of this information alongside clinical thresholds offers potential benefits for ease of interpretation and clinical decision-making.

Our study provides an analysis of critical limit and critical value test lists used by major US medical centers, revealing significant changes in quantitative critical limit thresholds since 1990 and documenting notification priorities for qualitative and new listings. Statistically significant differences were observed in clinical tests between 1990 and 2024, including glucose, calcium, magnesium, CO_2_ content, blood gas parameters, hematology, and coagulation tests. Cardiac biomarkers were infrequently listed, suggesting potential areas for improvement of standardization. POCT listings were sparse and showed a significant difference from main lab values in the high median critical limit for glucose. Only three pathogens—*Vibrio cholerae,* COVID-19, and the Influenza viruses—were consistently deemed as requiring urgent notification by the NIH, WHO, CDC, and IDSA.

Our research highlights the need for enhanced collaboration between government agencies and medical centers to establish universally accepted lists of critical values. Collaboration could lead to more consistent and timely patient care and ultimately improve patient outcomes. Future research should focus on developing standardized protocols for updating critical value lists and exploring the impact of these changes on patient care quality and efficiency.

## Figures and Tables

**Table 1:** Clinical chemistry critical limits.

Measurand	Listing Frequency (%)		Units	Low Mean (SD)		Low Median (Range)		High Mean (SD)		High Median (Range)	
	2024	1990		1990	2024	1990	2024	1990	2024	1990	2024
Glucose	100	100	mmol/L	2.6 (0.4)	2.7 (0.3)	**2.5** **(1.7–** **3.9)**	2.8 ** **(2.2–** **3.3)**	26.9 (8.0)	26.4 (2.8)	27.8 (6.1–55.5)	27.8 (22.1–33.3)
			mg/dL	46 (7)	49 (5)	**45** **(30–70)**	50 ** **(39–60)**	484 (144)	476 (51)	501 (110–1000)	500 (399–1000)
Calcium	98	100	mmol/L	1.65 (0.17)	1.55 (0.10)	**1.62** **(1.2–2.2)**	1.50 ** **(1.2–1.7)**	3.22 (0.22)	3.24 (0.15)	**3.24** **(2.62–3.49)**	3.24 ** **(2.87–3.49)**
			mg/dL	6.6 (0.7)	6.2 (0.4)	**6.5** **(5.0–8.6)**	6.0 ** **(5.0–7.0)**	12.9 (0.9)	13.0 (0.6)	**13.0** **(10.5–14.0)**	13.0 ** **(11.5–14.0)**
Sodium	98	100	mmol/L	120 (5)	121 (2)	120 (110–137)	120 (119–125)	158 (6)	159 (2)	160 (145–170)	160 (153-165)
Potassium	96	100	mmol/L	2.8 (0.3)	2.8 (0.2)	2.7 (2.5–3.6)	2.8 (2.5–3.0)	6.2 (0.4)	6.1 (0.2)	6.0 (5.0–8.0)	6.0 (5.9–7.0)
Magnesium	88	33	mmol/L	0.41 (0.16)	0.41 (0.04)	0.41 (0.21–0.74)	0.41 (0.25–0.49)	2.02 (0.82)	2.3 (0.66)	**1.75 (1.03–5.02)**	2.02 ** (1.44–4.53)
Ionized Calcium	82	57	mmol/L	0.82 (0.14)	0.78 (0.06)	0.80 (0.50–1.07)	0.80 (0.50–0.90)	1.55 (0.19)	1.59 (0.17)	1.50 (1.30–2.00)	1.58 (1.40–2.50)
Phosphorus	78	33	mmol/L	0.39 (0.10)	0.36 (0.06)	0.32 (0.26–0.65)	0.32 (0.29–0.48)	2.87 (0.48)	3.13 (0.48)	3.22 (2.26–3.23)	2.91 (2.58–4.04)
			mg/dL	1.2 (0.3)	1.1 (0.2)	1.0 (0.8–2.0)	1.0 (0.9–1.5)	8.9 (1.5)	9.7 (1.5)	10.0 (7.0–10.0)	9.0 (8.0–12.5)
CO_2_ Content	74	75	mmol/L	11 (2)	11 (2)	10 (5–20)	10 (8–15)	40 (3)	42 (3)	**40** **(35–50)**	40 * **(39–50)**
Lactate	62	5	mmol/L	…	…	…	…	3.4 (1.3)	3.9 (1.1)	3.0 (2.3–5.0)	4.0 (2.0–8.0)
Osmolality	40	20	mmol/kg	250 (13)	245 (14)	250 (230–280)	250 (190–260)	326 (18)	332 (18)	320 (295–375)	330 (308–390)
Cerebrospinal Fluid Glucose	40	16	mmol/L	2.1 (0.6)	1.9 (0.4)	2.2 (1.1–2.8)	2.2 (1.1–2.5)	24.3 (11.4)	17.4 (3.5)	16.7 (13.9–38.9)	16.7 (13.9–25.0)
			mg/dL	37 (10)	34 (8)	40 (20–50)	39 (20–50)	438 (206)	314 (63)	301 (250–700)	300 (250–450)
Ammonia	32	…	μmol/L	…	…	…	…	…	127 (57)	…	108 (50–250)
Chloride	22	20	mmol/L	75 (8)	77 (3)	80 (60–90)	75 (70–81)	126 (12)	125 (5)	120 (115–156)	125 (119–130)
Urea Nitrogen	12	20	mmol/L	…	…	…	…	37.1 (21.1)	32.5 (10)	33.9 (14.3–107.1)	36.1 (14.3–42.9)
			mg/dL	…	…	…	…	104 (59)	91 (28)	95 (40–300)	101 (40–120)
Creatinine	12	10	µmol/L	…	…	…	…	654 (380)	636 (265)	884 (177–1326)	663 (177–884)
			mg/dL	…	…	…	…	7.4 (4.3)	7.2 (3.0)	10 (2.0–15.0)	7.5 (2.0–10.0)
Uric Acid	8	20	µmol/L	…	…	…	…	773 (119)	714 (59)	892 (595–892)	773 (595–773)
			mg/dL	…	…	…	…	13 (2)	12 (1)	15 (10–15)	13 (10–13)
Bilirubin	0	25	µmol/L	…	…	…	…	257 (86)	ACNL	257 (86–513)	ACNL
			mg/dL	…	…	…	…	15 (5)	ACNL	15 (5–30)	ACNL

ACNL, Adult Critical limits Not Listed. *, Significant difference in medians at *p* < 0.05; **, Significant difference in medians at *p* < 0.01. Bold indicates statistically significant difference.

**Table 2:** pH and blood gas critical limits.

Measurand	Listing Frequency (%)		Units	Low Mean (SD)		Low Median (Range)		High Mean (SD)		High Median (Range)	
	2024	1990		1990	2024	1990	2024	1990	2024	1990	2024
pH	86	35	pH	7.21 (0.06)	7.20 (0.03)	7.20 (7.00–7.35)	7.20 (7.10–7.25)	7.59 (0.03)	7.59 (0.02)	7.60 (7.50–7.65)	7.60 (7.55–7.65)
Arterial pO_2_	78	26	mmHg	43(6)	45(6)	**40** **(30–55)**	40 * **(39–60)**	…	…	…	…
			kPa	5.7 (0.8)	6.0 (0.8)	**5.3** **(4.0–7.3)**	5.3 * **(5.2–** **8.0)**	…	…	…	…
Arterial pCO_2_	74	30	mmHg	19(3)	21(6)	**20** **(9–25)**	20 * **(9–44)**	67 (6)	67 (5)	70 (50–80)	70 (55–80)
			kPa	2.5 (0.4)	2.8 (0.8)	**2.7** **(1.2–** **3.3)**	2.7 * **(1.2–** **5.9)**	8.9 (0.8)	8.9 (0.7)	9.3 (6.7–10.7)	9.3 (7.3–10.7)
Carboxy- hemoglobin	48	…	%	…	…	…	…	…	15.3 (5.7)	…	17.0 (5.1–25.0)
Met- hemoglobin	34	…	%	…	…	…	…	…	11.7 (10.4)	…	5.1 (3.0–30.0)
Venous pH	26	…	pH	…	7.20 (0.04)	…	7.20 (7.10–7.25)	…	7.59 (0.03)	…	7.60 (7.52–7.60)
Venous pCO_2_	16	…	mmHg	…	20(6)	…	20 (9–28)	…	69(6)	…	68 (60–75)
			kPa	…	2.7(0.8)	…	2.7 (1.2–3.7)	…	9.2 (0.8)	…	9.1 (8.0–10.0)

*, Significant difference in medians at *p* < 0.05. Bold indicates statistically significant difference.

**Table 3:** Hematology and coagulation critical limits.

Measurand	Listing Frequency (%)		Units	Low Mean (SD)		Low Median (Range)		High Mean (SD)		High Median (Range)	
	2024	1990		1990	2024	1990	2024	1990	2024	1990	2024
Platelets	96	45	10⁹/L	37 (21)	25 (11)	**30 (10–100)**	20 ** (10–50)	894 (206)	984 (73)	1000 (100–1000)	1000 (600–1000)
Hemoglobin	96	42	g/dL	7.0 (2.2)	6.3 (0.6)	7.0 (4.0–15.0)	6.0 (5.0–7.0)	19.0 (4.4)	20.2 (0.9)	**20.0** **(20.0–30.0)**	**20.0** † **(19.0–22.5)**
INR	94	…	INR	…	…	…	…	…	4.9 (0.6)	…	5.0 (4.0–7.0)
Partial Thromboplastin Time	90	33	Sec	50	…	50	…	80 (33)	116 (25)	**80 (40–150)**	110 ** (69–200)
Fibrinogen	88	27	mg/dL	85 (26)	88 (19)	100 (8–100)	100 (50–120)	780 (260)	…	800 (500–1000)	…
WBC Count	84	42	10⁹/L	2.2 (0.9)	1.3 (0.5)	**2.0 (1–5)**	1.4 ** (0.5–2.0)	37 (22)	57 (31)	**30 (9.5–100)**	50 ** (25–150)
Hematocrit	72	29	%	20 (8.1)	19 (2)	18 (41)	20 (15–21)	60 (12.8)	61 (5)	60 (20–80)	60 (54–75)
Absolute Neutrophil Count	34	…	10⁹/L	…	0.6 (0.2)	…	0.5 (0.3–1.0)	…	…	…	…
WBC Count in CSF	26	…	WBC/mm^3^	…	…	…	…	…	39 (55)	…	20 (5–200)
Prothrombin Time	16	33	Sec	12 (2.4)	…	10 (10–15)	…	**29 (8)**	48 ** (5)	30 (14–40)	47 (40–58)
Band Count	12	…	%	…	…	…	…	…	23 (11)	…	20 (10–40)
Blast Cells	12	…	%	…	…	…	…	…	5.7 (2.2)	…	5 (4–10)

**, significant difference in medians at *p* < 0.01. †, 0.10 < *p* < 0.05. Bold indicates statistically significant difference.

**Table 4:** Newborn critical limits.

Test	Listing Frequency (%)		Units	Low Mean (SD)		Low Median (Range)		High Mean (SD)		High Median (Range)	
	2024	1990		1990	2024	1990	2024	1990	2024	1990	2024
**Clinical Chemistry**											
Bilirubin	94	9	µmol/L	…	…	…	…	239 (34)	257 (34)	257 (171–308)	257 (171–342)
			mg/dL	…	…	…	…	14 (2)	15 (2)	15 (10–18)	15 (10–20)
Glucose	80	42	mmol/L	1.8 (0.4)	2.2 (0.3)	**1.7 (1.1–2.8)**	2.2 ** (1.7–3.1)	18.1 (6.0)	14.7 (4.4)	16.7 (11.1–38.9)	13.9 (8.3–27.8)
			mg/dL	32 (7)	40 (6)	**31 (20–50)**	40 ** (30–55)	326 (108)	265 (80)	301 (200–700)	250 (150–500)
Potassium	30	32	mmol/L	2.6 (0.2)	2.8 (0.3)	**2.5 (2.5–3.5)**	2.9 ** (2.0–3.0)	7.7 (0.7)	6.8 (0.6)	**8.0 (5.5–8.0)**	6.9 ** (6.0–8.0)
Sodium	18	…	mmol/L	…	125 (2)	…	125 (124–130)	…	154 (4)	…	155 (150–160)
**Hematology**											
Hemoglobin	44	7	g/dL	9.5 (3.5)	7.9 (1.8)	8.5 (5.0–15.0)	8.0 (6.0–12.0)	22.3 (2.3)	22.4 (1.5)	21.0 (21.0–25.0)	22.0 (20.0–25.0)
Hematocrit	32	7	%	33 (6)	24 (6)	**30 (24–45)**	21 * (18–35)	**71 (4)**	66 * (3)	70 (65–75)	66 (60–70)
Platelets	28	…	10⁹/L	…	44 (22)	…	45 (20–100)	…	950 (84)	…	1000 (800–1000)
**Blood Gas and pH**											
pH Cord Blood	28	…	pH Units	…	7.05 (0.09)	…	7.00 (7.00–7.25)	…	7.52 (0.03)	…	7.50 (7.50–7.55)
Arterial pO_2_	16	8	mm Hg	37 (7)	46 (5)	**35 (30–50)**	50 * (40–50)	92 (12)	120 (73)	100 (70–100)	95 (90–300)
			kPa	4.9 (0.9)	6.1 (0.7)	**4.7 (4.0–6.7)**	6.7 * (5.3–6.7)	12.3 (1.6)	16.0 (9.7)	13.3 (9.3–13.3)	12.7 (12.0–40.0)
Arterial pCO_2_	16	2	mm Hg	35 (7)	30	35 (30–40)	30	55 (7)	66 (11)	55 (50–60)	70 (50–80)
			kPa	4.7 (0.9)	4.0	4.7 (4.0–5.3)	4.0	7.4 (0.9)	8.8 (1.5)	7.4 (6.7–8.0)	9.3 (6.7–10.7)

*, significant difference in medians and one mean at *p* < 0.05; **, significant difference in medians at *p* < 0.01. Bold indicates statistically significant difference.

**Table 5:** Cardiac biomarker listings.

Measurand	Listing Frequency (%)	Units	High Mean (SD)	High Median (Range)
Troponin I	24	ng/mL	0.59 (1.33)	0.30 (0.015–5.00)
High-Sensitivity Troponin I	20	ng/mL	0.16 (0.26)	0.075 (0.017–1.00)
Creatine Kinase (CK)	12	Units/L	4492 (4598)	3100 (250–10,000)
Female Troponin I	12	ng/mL	0.041 (0.028)	0.035 (0.015–0.075)
Male Troponin I	12	ng/mL	0.061 (0.036)	0.056 (0.02–0.10)
“Troponin”	12	ng/mL	0.45 (0.54)	0.28 (0.04–1.50)
CK-MB	6	ng/mL	7.1 (0.85)	7.0 (6.3–8.0)
Troponin T	6	ng/mL	0.20 (0.17)	0.10 (0.10–0.40)
High-sensitivity Troponin T	2	ng/mL	0.052	0.052
Brain natriuretic peptide (BNP)	2	pg/mL	1300	1300

**Table 6:** Listing frequencies of critical pathogens.

Pathogen	NIH	CDC	WHO	IDSA	Frequency (%)
*Cryptococcus* species (*C. neoformans* or *gatti*)					70
Herpes simplex virus (HSV)					50
Malarial parasites					42
Tuberculosis (*Mycobacterium tuberculosis*)					34
Human Immunodeficiency Virus (HIV)					32
Brucellosis (*Brucella* species)					26
Tularemia (*Francisella tularensis*)					26
Anthrax (*Bacillus anthracis*)					24
Plague (*Yersinia pestis*)					24
*Pneumocystis* species					24
*Coccidiodes* species					22
*Burkholderia mallei* or *psuedomallei*					20
*Bordatella* *pertussis*					18
*Escherichia coli*					18
Legionella					18
*Neisseria meningitidis*					18
Varicella Zoster Virus (VZV)					18
Syphilis					16
Botulism *(Clostridium botulinum* toxin)					14
Cholera (*Vibrio cholerae*)	**Consensus**				14
Methicillin-resistant *Staphylococcus aureus* (MRSA)					14
*Salmonella* species					14
Viral hemorrhagic fever					8
Influenza viruses	**Consensus**				6
Smallpox (Variola minor)					6
COVID-19 (SARS-CoV-2)	**Consensus**				4

Gray fill-ins indicated that the corresponding pathogen is deemed as demanding rapid detection by that agency.

**Table 7:** Qualitative critical values.

Measurand	Detection Method(s) (If Identified)	Listing Frequency (%)
A. Microbiology		
Positive culture from blood, cerebrospinal fluid, or sterile body fluid	Culture	82
*Cryptococcus* species (*Cryptococcus neoformans* or *gattii*)	Culture, Latex agglutination, PCR, RAgT	70
Acid-Fast Bacillus (AFB)	Culture, stain	68
Positive Gram stain from blood, cerebrospinal fluid, or sterile body fluid	Gram stain	60
Pathogenic fungus in a potentially invasive space (CSF, blood, or sterile body fluid)	Culture, PCR, smear, stain	44
*Mycobacterium tuberculosis*	Culture, PCR, stain	34
Dimorphic fungal pathogens (e.g., *Histoplasma*, *Blastomyces*, or *Coccidioides* species)	Culture, PCR, smear	32
Brucellosis (*Brucella* species)	Culture	26
Tularemia (*Francisella tularensis*)	Culture	26
Anthrax (*Bacillus anthracis*)	Culture	24
Antibiotic resistant bacteria	Culture	24
Eye and ocular cultures positive	Culture, Gram stain	24
Plague (*Yersinia pestis*)	Culture	24
*Pneumocystis* species	PCR, RAgT	24
*Burkholderia mallei* or *pseudomallei*	Culture	20
*Bordetella pertussis*	Culture, PCR	18
*Escherichia coli* O157:H7 or Shiga-toxin tests	Culture, PCR	18
Legionella	Culture, RAgT	18
*Neisseria meningitidis* from sterile sites	Culture, RAgT	18
Meningitis/Encephalitis panel	PCR	16
Syphilis	RPR	16
Botulism (*Clostridium botulinum* toxin)	Culture	14
*Candida auris*	Culture, PCR	14
Cholera (*Vibrio cholerae*)	-	14
*Corynebacterium diphtheriae*	Culture	14
Group A *Streptococcus*	Culture, RAgT	14
Listeria	Culture	14
Methicillin-resistant *Staphylococcus aureus* (MRSA)	-	14
*Neisseria gonorrhea* in the eye	Culture, probe	14
*Salmonella* species	Culture	14
Vancomycin Resistant *Enterococcus* (VRE)	-	14
*Pseudomonas* eye/ocular positive	Culture, probe	12
*Streptococcus pneumoniae*	Culture, PCR, RAgT	12
Group B *Streptococci*	Culture, PCR, RAgT	10
*Shigella*	Culture	10
Vancomycin-resistant *Staphylococcus aureus* (VRSA)	-	10
Chlamydia	Culture, PCR	8
*Clostridium difficile*	Culture, PCR	8
Nocardia	Culture, PCR	8
Q fever (*Coxiella burnetii*)	-	8
B. Virology		
Herpes simplex virus (HSV) in CSF	PCR	36
Herpes simplex virus (HSV) in newborns or near-term pregnant mothers	Culture, Pap, PCR	34
Human Immunodeficiency Virus (HIV)	Ag/Ab combo, PCR, RAgT, Western blot	32
Varicella zoster virus (VZV)	PCR	18
Epstein–Barr Virus (EBV)	PCR	14
Cytomegalovirus (CMV)	Culture, PCR	12
Respiratory Syncytial Virus (RSV)	PCR	12
Enterovirus in blood/sterile sites	Culture, PCR	10
Hepatitis B surface antigen	-	8
Viral hemorrhagic fever	-	8
Smallpox (Variola major)	-	6
COVID-19 (SARS-CoV-2) Detected	PCR, RagT	4
C. Parasitology		
Malarial parasites	RAgT, smear	42
Parasites from sterile body sites	RAgT, smear	22
*Babesia microti*	PCR, Smear	14
*Plasmodium* species	Smear, stain	10
Microfilaria	Smear, stain	8
D. Blood Bank		
Positive Direct Coombs test/Direct antiglobulin test (DAT)	-	28
Hemolytic transfusion reaction	-	22
Positive culture or Gram stain on a blood product associated with a transfusion reaction	Culture, Gram stain	20
New positive antibody screen	-	20
Non-availability of ordered transfusion	-	18
Incompatible crossmatch	-	16
Fetal maternal hemorrhage		8
E. Hematology		
Presence of blasts in blood or CSF	Smear	26
Heparin induced platelet antibodies	-	10
New diagnosis or findings of leukemia	Smear	8
F. Anatomical/Surgical Pathology		
Fat in endometrial curettage	-	14
Significant disagreement between the frozen section and final diagnosis	-	14
Significant discrepancy between outside diagnosis and the review diagnosis	-	14
Unexpected diagnosis of malignancy	-	14
Amended report with a significant change in diagnosis	-	12
Absence of chorionic villi when clinically expected	-	10
Crescents in kidney biopsy specimen	-	8
Significant discrepancy between the fine needle aspiration (FNA) immediate interpretation and the final diagnosis	-	8
Transplant rejection	-	8
Unexpected fat in colonic endoscopic specimens	-	8
G. Urinalysis		
Positive urine glucose	-	18
Positive urine ketones	-	16
RBC casts	Microscope	8

Abbreviations (in addition to those in the table): COVID-19, coronavirus infectious disease-2019; CSF, cerebrospinal fluid; PCR, Polymerase Chain Reaction; RAgT, rapid antigen test; RBC, red blood cells; RPR, rapid plasma reagin; and SARS-CoV-2, severe acute respiratory syndrome-coronavirus-2.

**Table 8:** POCT critical limits.

Measurands	Listing Frequency (%)	Units	Low Mean (SD)	Low Median (Range)	High Mean (SD)	High Median (Range)
**Clinical Chemistry**						
Glucose	24.1	mg/dL	49.5	50	415.4	400
			(4.4)	(40–55)	(87.5)	(200–500)
Newborn Glucose	9.3	mg/dL	40.8	40	220.0	200
			(7.3)	(30–50)	(73.7)	(125–325)
Potassium	16.7	mmol/L	2.6	2.5	6.2	6.0
			(0.3)	(2.0–3.0)	(0.2)	(6.0–6.5)
Ionized Calcium	14.8	mmol/L	0.81	0.77	1.57	1.50
			(0.10)	(0.70–1.0)	(0.13)	(1.40–1.75)
Sodium	14.8	mmol/L	121.1	120	160.1	160
			(2.1)	(120–125)	(2.4)	(156–165)
**Blood Gases and pH**						
pCO_2_	13.0	mm/Hg	20	20	64.3	70
			(3.2)	(15–25)	(7.9)	(50–70)
pH	13.0	na	7.21	7.20	7.59	7.60
			(0.04)	(7.2–7.3)	(0.019)	(7.55–7.6)
pO_2_	13.0	mm/Hg	45.7	45	na	na
			(6.1)	(0–55)		
**Hematology**						
Hematocrit	11.1	%	18.2 (2.1)	18 (15–21)	63.3 (6.1)	60 (60–75)
Hemoglobin	11.1	g/dL	6.4	6.25	20.3	20
			(0.5)	(6–7)	(0.5)	(20–21)
INR	9.3	na	na	na	4.9	5
					(0.2)	(4.5–5.0)

INR, International normalized ratio.

## Data Availability

The datasets presented in this article are not readily available due to confidentiality of sources and direct collection of many lists. Requests to access the datasets should be directed to geraldkost@gmail.com.

## Abbreviations

The following abbreviations are used in this manuscript:

POCT	Point-of-care testing
JAMA	*Journal of the American Medical Association*
MLO	*Medical Laboratory Observer*
CLR	Clinical Laboratory reference
CDC	Centers for Disease Control and Prevention
NIH	National Institutes of Health
WHO	World Health Organization
IDSA	Infectious Diseases Society of America
ADA	American Diabetes Association
ACNL	Adult Critical limits Not Listed
PTT	Partial thromboplastin time
PT	Prothrombin time
AMI	Acute myocardial infarction
WBC	White blood cell
ANC	Absolute neutrophil count
INR	International normalized ratio
CSF	Cerebrospinal fluid
TnI	Troponin I
hs-TnI	High-sensitivity Troponin I
CK	Creatine Kinase
AFB	Acid-Fast Bacillus
COVID-19	Coronavirus infectious disease-2019
PCR	Polymerase Chain reaction
RAgT	Rapid Antigen Test
RBC	Red blood cells
RPR	Rapid plasma reagin
SARS-CoV-2	Severe acute respiratory syndrome-coronavirus-2
