# Correction: Shah et al. Visualization of Critical Limits and Critical Values Facilitates Interpretation. *Diagnostics* 2025, *15*, 604

**DOI:** 10.3390/diagnostics16132057

**Published:** 2026-07-01

**Authors:** Ania Shah, Jenna Dohner, Kaifeng Cheng, Maria Garcia, Gerald J. Kost

**Affiliations:** 1 University of California, Davis, CA 95616, USA; 2 University Honors Program, University of California, Davis, CA 95616, USA; 3 Pathology and Laboratory Medicine, School of Medicine, University of California, Davis, CA 95616, USA

In the original publication [1], there were mistakes in Table 8. POCT critical limits, as published. Column entries on the right were interchanged and the units for ionized calcium were incorrect. The corrected Table 8 appears below. The authors state that the scientific conclusions are unaffected. This correction was approved by the Academic Editor. The original publication has also been updated.

## Figures and Tables

**Table 8:** POCT critical limits.

Measurands	Listing Frequency (%)	Units	Low Mean (SD)	Low Median (Range)	High Mean (SD)	High Median (Range)
**Clinical Chemistry**						
Glucose	24.1	mg/dL	49.5	50	415.4	400
			(4.4)	(40–55)	(87.5)	(200–500)
Newborn Glucose	9.3	mg/dL	40.8	40	220.0	200
			(7.3)	(30–50)	(73.7)	(125–325)
Potassium	16.7	mmol/L	2.6	2.5	6.2	6.0
			(0.3)	(2.0–3.0)	(0.2)	(6.0–6.5)
Ionized Calcium	14.8	mmol/L	0.81	0.77	1.57	1.50
			(0.10)	(0.70–1.0)	(0.13)	(1.40–1.75)
Sodium	14.8	mmol/L	121.1	120	160.1	160
			(2.1)	(120–125)	(2.4)	(156–165)
**Blood Gases and pH**						
pCO_2_	13.0	mm/Hg	20	20	64.3	70
			(3.2)	(15–25)	(7.9)	(50–70)
pH	13.0	na	7.21	7.20	7.59	7.60
			(0.04)	(7.2–7.3)	(0.019)	(7.55–7.6)
pO_2_	13.0	mm/Hg	45.7	45	na	na
			(6.1)	(0–55)		
**Hematology**						
Hematocrit	11.1	%	18.2 (2.1)	18 (15–21)	63.3 (6.1)	60 (60–75)
Hemoglobin	11.1	g/dL	6.4	6.25	20.3	20
			(0.5)	(6–7)	(0.5)	(20–21)
INR	9.3	na	na	na	4.9	5
					(0.2)	(4.5–5.0)

INR, International normalized ratio.
